# Antidiabetic Activity of Zinc Oxide and Silver Nanoparticles on Streptozotocin-Induced Diabetic Rats

**DOI:** 10.3390/ijms15022015

**Published:** 2014-01-28

**Authors:** Ali Alkaladi, Aaser Mohamed Abdelazim, Mohamed Afifi

**Affiliations:** 1 Department of Biological Sciences, Faculty of Science, King Abdulaziz University, North Campus, P.O. Box 11508, Jeddah 21463, Saudi Arabia; E-Mail: alkaladi@kau.edu.sa; 2 Department of biochemistry, Faculty of Veterinary Medicine, Zagazig University, Zagazig 44511, Egypt; E-Mail: drasr_bio@yahoo.com

**Keywords:** diabetes, ZnONPs, SNPs

## Abstract

The use of nanoparticles in medicine is an attractive proposition. In the present study, zinc oxide and silver nanoparticles were evaluated for their antidiabetic activity. Fifty male albino rats with weight 120 ± 20 and age 6 months were used. Animals were grouped as follows: control; did not receive any type of treatment, diabetic; received a single intraperitoneal dose of streptozotocin (100 mg/kg), diabetic + zinc oxide nanoparticles (ZnONPs), received single daily oral dose of 10 mg/kg ZnONPs in suspension, diabetic + silver nanoparticles (SNPs); received a single daily oral dose of SNP of 10 mg/kg in suspension and diabetic + insulin; received a single subcutaneous dose of 0.6 units/50 g body weight. Zinc oxide and silver nanoparticles induce a significant reduced blood glucose, higher serum insulin, higher glucokinase activity higher expression level of insulin, insulin receptor, *GLUT-2* and glucokinase genes in diabetic rats treated with zinc oxide, silver nanoparticles and insulin. In conclusion, zinc oxide and sliver nanoparticles act as potent antidiabetic agents.

## 1. Introduction

Diabetes mellitus is a metabolic disorder that characterized by high blood glucose. A large number of people suffer from diabetes all over the world [1]. These patients would require the development of several medications with multiple modes of actions. Many researches demonstrated the role of metals in glucose metabolism and the association of their deficiency with diabetes. Vanadium [2], chromium [3], magnesium [4], and zinc [5] have been reported to play a role in blood sugar maintenance and have been included in diabetes therapy. Zinc, an essential metal, is an activator for more than three hundred enzymes in the body [6], and plays a key role in different metabolic pathways including glucose metabolism. Zinc promotes hepatic glycogenesis through its actions on the insulin pathways and thus improves glucose utilization [7]. Zinc is also known to keep the structure of insulin [8] and has a role in insulin biosynthesis, storage and secretion [5]. There are several zinc transporters in pancreatic b cells [9]; like zinc transporter 8 which has a potent role in insulin secretion [10]. In addition, zinc could improve insulin signaling by several mechanisms, including increased insulin receptor phosphorylation, enhancing PI3K activity and inhibition of glycogen synthase kinase-3 [7]. The beneficial role of zinc in diabetes has been implicated by studies of the zinc supplies in diabetic rats [11]. Although silver is an important metal in a huge number of metabolic processes; there is no data about the effective power of silver or silver nanoparticles (SNP) on the glucose status. This encourages us to study the effect of SNP in comparison with ZnONPs.

## 2. Results and Discussion

**Figure 1.**
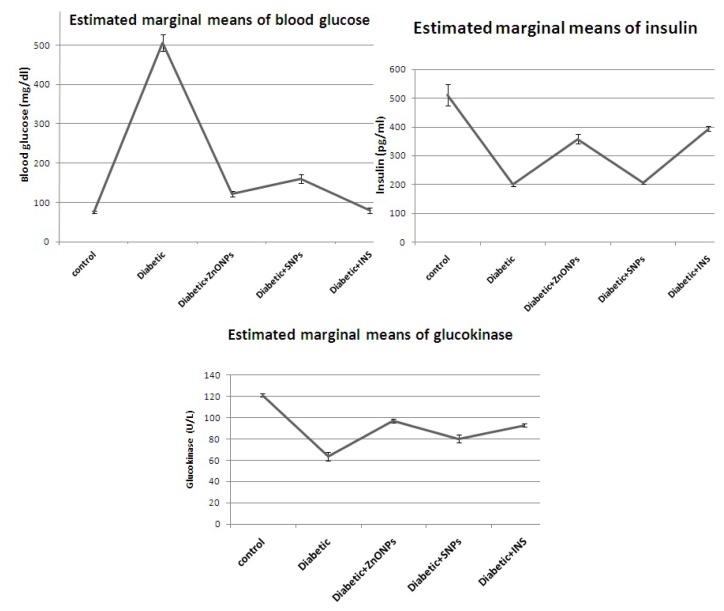
Estimated marginal means of blood glucose level (mg/dL), insulin level (pg/mL) and glucokinase activity (U/L).

**Figure 2.**
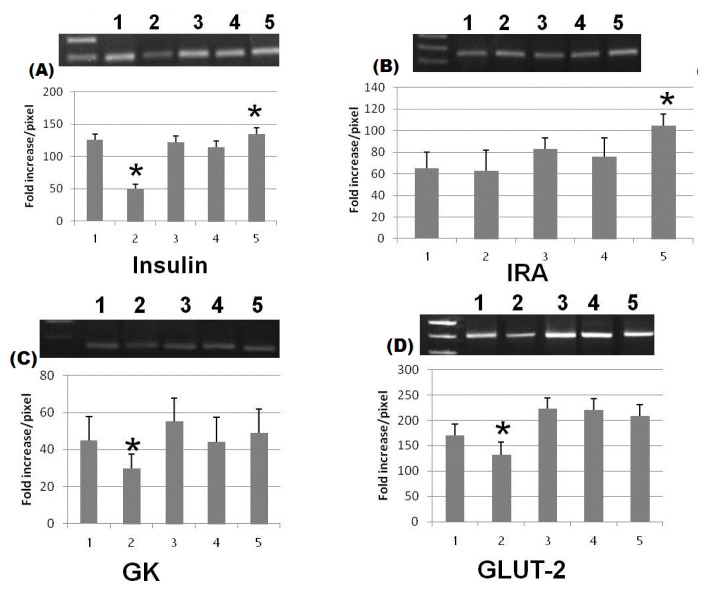

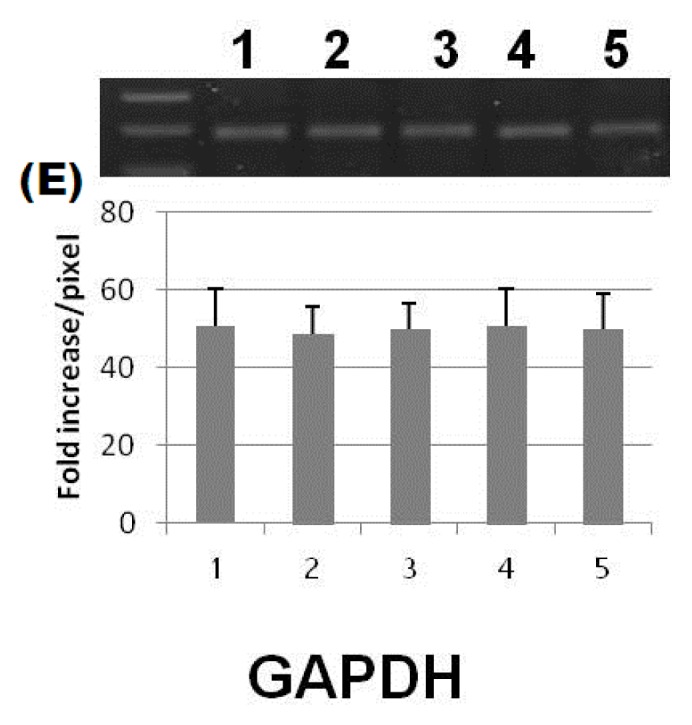
Gel picture of the examined genes bands after Agarose gel electrophoresis (**A**) Insulin gene; (**B**) Insulin receptor A gene; (**C**) Glucokinase gene; (**D**) Glucose transporter protein 2; and (**E**) Glycraldehyd 3 phosphate dehydrogenase. 1, control; 2, diabetic; 3, diabetic + ZnONPs; 4, diabetic + SNPs; and 5, diabetic + insulin (column with (*) are significant at *p* < 0.05).

**Figure 3.**
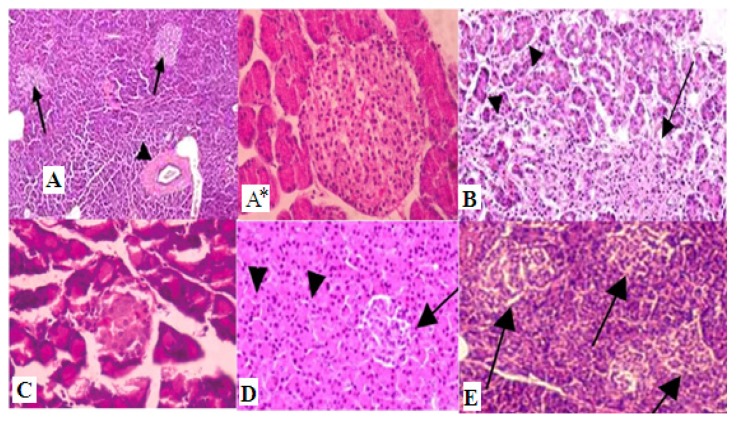
Histopathology graphs shows (**A**) Control, normal pancreas showing normal islets of Langerhans in between normal pancreatic acini (arrow) and normal pancreatic duct (arrow head); (A*) normal pancreatic acini (×100); (**B**) Diabetic pancreas showing ruptured and destructed islets of Langerhans with damage in B-cells; (**C**) Diabetic and ZnONP group, pancreas showing some normal islets of Langerhans with some destructed one in between normal pancreatic acini and normal pancreatic duct; (**D**) Diabetic and SNP rats pancreas showing ruptured and destructed islets of Langerhans with damage in B-cells (arrow); and (**E**) Diabetic insulin rats pancreas showing ruptured and destructed islets of Langerhans with damage in B-cells. Hematoxylin and Eosin (H&E) (×100).

In the present study we evaluated the possible therapeutic effect of zinc oxide and silver nanoparticles on streptozotocin-induced diabetic rats as well as their compared effect to insulin treatment. There are various kinds of nanoparticles that are evaluated for their use as drug delivery systems [12]. The metals nanoparticles, as zinc, silver, iron and gold, oxides of nanoparticles, have a great role in medical and biological applications [13]. Our results showed a great reduction in blood glucose level in diabetic groups treated with ZnONPs, SNPs and insulin (75.8%, 68.2% and 84.2%) respectively (Table 1). This showed a great antidiabetic activity of those nanoparticles. However, ZnONPs induce more reduction than SNP as zinc has been elucidated to be a potent metal that improves glucose utilization and metabolism through its potent influence on enhancement of hepatic glycogenesis through actions on the insulin signaling pathway [7]. Our experiments revealed that ZnONPs and SNP could increase serum insulin level in diabetic groups treated with ZnONPs and SNP (79.4% and 3%) respectively if compared with diabetic groups treated with insulin (97.3%). It appeared that ZnONPs also induced more insulin secretion if compared to the effect of SNPs (Figure 1). In addition, the mRNA expression level of insulin gene appeared to increase in ZnONPs and insulin treated groups if compared with the diabetic non-treated group (Figure 2). There are few studies that have investigated the therapeutic effect of ZnONPs on insulin levels or secretion. However, others have demonstrated that zinc could enhance the glucose stimulated insulin secretion from rat isolated pancreatic islets [14]. Interestingly, on the basis of Umrani and Paknikar, [15], ZnONPs did not possess the risk of hypoglycemia in living organisms so it can act as an insulin secretagog. Similarly, SNPs could also increase the insulin level but by a very low percentage (3%) if compared with ZnONPs; this may be due to accumulation of zinc in the secretory vesicle of B cells using transporter 8 [10,16]. Zinc transporters are also identified in adipose tissues and liver [17]. Such organs are the major regulator of glucose metabolism. All tissues that respond to insulin are able to express insulin receptors (IRs) with different levels and the ability of insulin to perform its function need to bind to alfa-subunits of its receptors has lead to the phosphorylation of β-subunits [18]. This supports our experimental results which showed high expression levels of *IRA* gene in hepatic tissues in the groups treated with ZnONPs and SNPs (Figure 2). Glucokinase (GK) activity was determined in the livers of all experimental rats, there is an increase in the activity of GK in ZnONPs, SNPs and insulin treated groups (52.5%, 25.8% and 44.7%) respectively if compared with diabetic non treated groups (Table 1). It appeared that the ZnONPs treated group showed the highest activity among treated groups. In addition, the expression level of the *GK* gene is proportional with its activity in all experimental rats. GK catalyzes the first step of glucose utilization in the liver and has a very low affinity to glucose in blood. This property makes it very sensitive to any change in blood glucose level [19]. It is well-known that the expression level and activity of GK are declined in diabetic models [20]. It is possible, therefore, that liver *GK* gene expression may decrease with the development of diabetes. On the other hand, activation of GK enhances the uptake of glucose by liver and pancreatic insulin secretion and, therefore, it is a strong marker for diabetic therapy [21]. The expression level of *GLUT-2* gene was determined in hepatic tissue. *GLUT-2* is membrane-bound and thus is not translocated by insulin. *GLUT-2* has a high Michaelis constant (*K*m) for glucose so that glucose transport into the liver is not limited [22]. Altered expression of *GLUT-2* gene has been studied particularly in relation to the pathogenesis of diabetes. Reduced *GLUT-2* gene expression is found in association with defective glucose-stimulated insulin release in animal models of diabetes [23]. Our data showed that ZnONPs and SNPs have the ability to increase the expression level of *GLUT-2* in hepatic tissues (Figures 2 and 3). This has lead to the enhancement of uptake and release of glucose from hepatocytes [24].

## 3. Experimental Section

### 3.1. Animal Selection and Grouping

Fifty male albino (Sprague-Dawley) rats, with average age and weight at the beginning of the experiment (6 months and 120 ± 20 g). All experimental animals were housed together for 7 days before the beginning of the experiment. Animals were randomly classified into five groups; the first group served as a control; these animals did not receive any type of treatment, 40 animals received a single intraperitoneal dose of streptozotocin (Sigma-Aldrich, Catalog No S0130 SIGMA, Seelze, Germany) with a dose equals 100 mg/kg [25] for induction of diabetes. They were further classified into four more groups; diabetic groups; they did not receive any type of treatment, diabetic + ZnONPs groups; received oral daily dose of ZnONPs (Sigma-Aldrich, Catalog No.721077, Seelze Germany) of 10 mg/kg for 30 constitutive days, diabetic + SNPs group; received oral daily dose of SNP (Sigma-Aldrich, Catalog No. 730793, Seelze, Germany) of 10 mg/kg for 30 constitutive days and diabetic + insulin group; received subcutaneous dose of insulin 0.6 units/50 g.

### 3.2. Animal Management

Rats of different groups were housed in different polypropylene cages. Standard laboratory pellet feed and purified drinking water was provided *ad libitum*. The temperature of the experimental room was maintained at 22 ± 3 °C. Relative humidity was controlled to be within 70% and a 12 h light/12 h dark cycle was maintained. Oral dosing was carried out using an 18-gauge oral feeding needle.

### 3.3. Ethical Statement

All experiments were carried out in accordance with the Saudi Arabian laws and University guidelines for the care of experimental animals. All procedures of the current experiment have been approved by the Committee of the Faculty of Science, North Jeddah, King Abdulaziz University, Jeddah, Saudi Arabia.

### 3.4. Induction of Experimental Diabetes

Experimental diabetes mellitus was induced by injection a single intraperitoneal dose of STZ (Sigma Chemial Co., Poole, Dorst, UK) equals 100 mg/kg in 0.01 M sodium citrate buffer (pH = 4.5). The rats were fed high sucrose and high fat diet (10% margarine, 20% sucrose and 65.5% basal rat diet). Forty rats were injected with freshly prepared STZ. After injection of (STZ), fasting blood glucose was determined and the rats with blood glucose level over 250 mg/dL were indicated as diabetic and grouped in diabetic groups.

### 3.5. Sampling Protocol

Blood samples were collected from a tail vein in all experimental rats. Serum (~150 μL) was separated by centrifugation, stored at −20 °C and analyzed later for blood glucose and insulin levels. Liver tissues were collected and homogenized for Glucokinase activity. Liver and spleen tissues for molecular investigations were taken on liquid nitrogen until isolation of genomic RNA.

### 3.6. Biochemical Determinations

Blood glucose (mg/dL) was estimated by glucose oxidase method using the kit supplied by SPINREACT (Sant Esteva de Bas, Girona, Spain) (http://www.spinreact.com.mx/public/_pdf/1001190.pdf), we measured blood glucose in all experimental animals before the beginning of the experimental procedures, after streptozotocin injection. For targeted-induced diabetic animals; blood glucose was routinely measured until diabetes was detected (animals with blood glucose >250 mg/dL are indicted as diabetics). After that, blood glucose was monitored in all experimental animals, and results were obtained at the end of the experimental period. Serum insulin level was estimated using a rat insulin elisa kit (Catalog No. ezrmi-13kelisa, EMD Millipore, Billerica, MA, USA) and liver tissue Glucokinase (GK) activity was determined according to the method described by Pakoskey *et al.* [26].

### 3.7. Molecular Biological Determinations

Total RNA will be extracted from liver tissues; using an RNA extraction Kit (AXYGEN, Biosciences, Central Avenue, Union city, CA, USA). First strand cDNA was synthesized using RevertAidTM H Minus (Fermentas, life science, Pittsburgh, PA, USA). Each RT reaction contained RNA template (3 μg/μL), 200 U/μL RevertAidTM H Minus M-MuL V Reverse Transcriptase (Fermentas, life science, Pittsburgh, PA, USA), 100 μM, 0.2 μg/μL random hexamer primers, 10 mM dNTPs mix, 20 U/μL RiboLock™ RNase inhibitor (Fermentas, Life Science, Pittsburgh, PA, USA), and 5× reaction buffer (250 mM Tris-HCl (pH 8.3), 250 mM KCl, 20 mM MgCl_2_, 50 mM DTT). Primers for all examined genes were chosen according to the following parameters: primer length within 20 bases; GC content 55%. The PCR reaction was started by using (DreamTaqTM Green PCR Master Mix (2×), Fermentas, Life Science, Pittsburgh, PA, USA). The reaction was performed using thermal cycler (Biosystems, Carlsbad, CA, USA). The primer pairs for amplification were designed as the following, insulin primers; forward, 5′-ATG GCC CTG TGG ATG CGC TT-3′ and reverse, 5′-TAG TTG CAG TAG TTC TCC AGC T-3′, insulin receptor primers; forward, 5′-TTC ATT CAG GAA GAC CTT CGA-3′ and reverse, 5′-AGG CCA GAG ATG ACA AGT GAC-3′, Glucose transporter-2 primers; forward, 5′-TTA GCA ACT GGG TCT GCA AT-3′ and reverse, 5′-TCT CTG AAG ACG CCA GGA AT-3′, Gk primers; forward, 5′-CAC CCA ACT GCG AAA TCA CC-3′and reverse, 5′-CAT TTG TGG GGT GTG GAG TC-3′and for GAPDH, forward, 5′-CCC GTA GAC AAA ATG GTG AAG GTC-3′and reverse, 5′-GCC AAA GTT GTC ATG GAT GAC C-3′ with product sizes, 331, 222, 243, 162 and 215 bp respectively. Amplified PCR products were then electrophorised on 1.5% Agarose gel in 1× Tris acetate EDTA running buffer (1 × TAE) with condition of 100 V/40 min. Samples were visualized using UV transilluminator T2621BS, (BioRad, Berkeley, CA, USA).

### 3.8. Histopathological Examinations

Specimens from the pancreas were collected and fixed in 10% buffered neutral formalin solution, dehydrated in gradual ethanol (70%–100%), cleared in xylene, and embedded in paraffin. Five-micron thick paraffin sections were prepared and then routinely stained with hematoxylin and eosin (HE) dyes [27] and then examined microscopically.

### 3.9. Statistical Analyses

All data are presented and analyzed using GraphPad Prism^®^ software (GraphPad Software, Inc., La Jolla, CA, USA). All results are expressed as mean ± standard error (SE). Comparison among groups was made by Student’s *t*-test (unpaired), One-way analysis of variance (ANOVA). Duncan’s test was used for testing the inter-grouping homogeneity. Statistical significance was set *p* < 0.05.

## 4. Conclusions

ZnONPs and SNPs were elucidated as antidiabetic agents. Although we concluded that ZnONPs are more powerful in their effect than silver nanoparticles, they all lead to reduction of blood glucose, increased insulin level and expression, increased GK activity and expression and improved expression level of *IRA*, *GLUT-2* in diabetic rats.

## Figures and Tables

**Table 1.** Biochemical effect of zinc oxide, silver nanoparticles and insulin treatment on streptozotocin-induced diabetic rats.

Parameters	Control	Diabetic	Diabetic + ZnONPs	Diabetic + SNP	Diabetic + insulin
Blood glucose (mg/dL)	76.4 ± 2.48 ^d^	505 ±2 1.04 ^a^	122.2 ± 6.62 ^c^	160.6 ± 10.56 ^b^	80.2 ± 6.36 ^d^
Insulin (pg/mL)	512 ± 36.93 ^a^	199.9 ± 4.93 ^c^	358.6 ± 16.46 ^b^	206.2 ± 3.33 ^c^	394.4 ± 7.21 ^b^
Glucokinase (U/L)	121 ± 1.51 ^a^	63.6 ± 3.83 ^d^	97 ± 1.58 ^b^	80.2 ± 3.86 ^c^	92.8 ± 1.48 ^b^

Means within the same raw carrying different superscripts (a, b, c, and d) are significant at *p* ≤ 0.05.
